# Ultraviolet Light Provides a Major Input to Non-Image-Forming Light Detection in Mice

**DOI:** 10.1016/j.cub.2012.05.032

**Published:** 2012-08-07

**Authors:** Floor van Oosterhout, Simon P. Fisher, Hester C. van Diepen, Thomas S. Watson, Thijs Houben, Henk Tjebbe VanderLeest, Stewart Thompson, Stuart N. Peirson, Russell G. Foster, Johanna H. Meijer

**Affiliations:** 1 Laboratory for Neurophysiology, Department of Molecular Cell Biology, Leiden University Medical Center, PO Box 9600 Mailbox S5-P, 2300 RC Leiden, The Netherlands; 2 Nuffield Laboratory of Ophthalmology, Nuffield Department of Clinical Neurosciences, Levels 5-6 West Wing, University of Oxford, John Radcliffe Hospital, Headley Way, Oxford OX3 9DU, UK; 3 Department of Ophthalmology and Visual Sciences, University of Iowa, 200 Hawkins Drive, Iowa City, IA 52242-1091, USA

## Abstract

The change in irradiance at dawn and dusk provides the primary cue for the entrainment of the mammalian circadian pacemaker. Irradiance detection has been ascribed largely to melanopsin-based phototransduction [1–5]. Here we examine the role of ultraviolet-sensitive (UVS) cones in the modulation of circadian behavior, sleep, and suprachiasmatic nucleus (SCN) electrical activity. UV light exposure leads to phase-shifting responses comparable to those of white light. Moreover, UV light exposure induces sleep in wild-type and melanopsin-deficient (*Opn4*^*−/−*^) mice with equal efficacy. Electrical recordings from the SCN of wild-type mice show that UV light elicits irradiance-dependent sustained responses that are similar to those induced by white light, with characteristic fast transient components occurring at the light transitions. These responses are retained in *Opn4*^*−/−*^ mice and preserved under saturating photopic conditions. The sensitivity of phase-shifting responses to UV light is unaffected by the loss of rods but is severely attenuated by the additional loss of cones. Our data show that UVS cones play an important role in circadian and sleep regulation in mice.

## Highlights

► UV light induces phase shifts in an irradiance-, duration-, and time-dependent manner ► UV light induces sleep in wild-type and melanopsin-deficient (*Opn4*^−/−^) mice ► UV light induces sustained intensity-dependent responses in the SCN of *Opn4*^−/−^ mice ► *rd/rd cl* mice show a severe attenuation in phase-shifting responses to UV light

## Results and Discussion

### Behavioral Responses to UV Light

**Figure 1:**
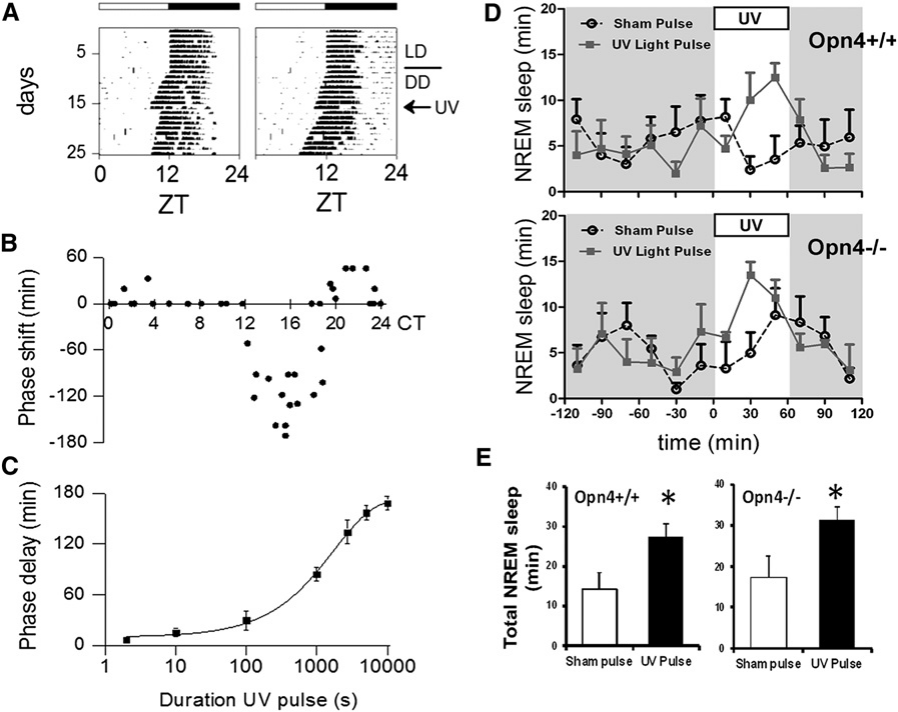
Behavioral and NREM Sleep Responses to UV Light (A) Representative actograms showing the phase-shifting response of wheel running activity to UV light in C57BL/6 mice. UV light pulses were applied on the seventh day in continuous darkness (DD) (CT15). (B) Phase response curve (PRC) of wheel running activity to UV light pulses (365 nm, 12.9 log quanta/cm^2^/s, 45 min exposure). Phase-shift magnitude and direction are plotted as a function of the circadian time. (C) Duration effects of phase shifts in response to UV light exposure at CT16 (365 nm, 12.9 log quanta/cm^2^/s). Phase shifts are duration dependent, increasing in magnitude with longer light exposure as has been previously shown for white light. Data points indicate mean ± SEM. (D) Time course of nonrapid eye movement (NREM) sleep following UV light exposure, showing mean responses ± SEM in *Opn4*^*+/+*^ and *Opn4*^*−/−*^ mice (n = 5). (E) Histograms summarizing changes in NREM sleep ± SEM in response to UV light exposure. UV light administered at zeitgeber time 16–17 resulted in a significant increase in NREM sleep in *Opn4*^*+/+*^ and *Opn4*^*−/−*^ mice. ^∗^p < 0.05. See also Figure S1.

We assessed the phase-shifting effects of UV light on circadian wheel running activity (Figures 1A–1C). Wild-type mice show a robust response to UV light (Figure 1A), and the full phase response curve to UV light (UV LED, 12.9 log quanta/cm^2^/s, 45 min) is shown in Figure 1B. Phase delays during the early subjective night (CT15 ± 1.5 hr) were −127 ± 11 min, and small but significant advances occurred in the late subjective night (mean shift ± SEM at CT21 ± 1.5 hr = 29 ± 8 min). No effect of UV light was found during the subjective day (CT0–CT12). These phase delays and small advances mirror those for white light in C57BL/6 mice [6, 7] and of UV light in the field mouse [8].

The effect of light duration on the magnitude of phase shifts in wheel running activity was investigated at the time of maximal phase delays (CT14–CT16; Figure 1C). Pulses of up to 10 s did not induce significant phase shifts (2 s: 6.8 ± 2.3 min; 10 s: 15.3 ± 4.4 min, p > 0.05). Following 100 s pulses, however, the phase of wheel running activity onset was significantly delayed (29.6 ± 10.9 min, p < 0.05). In response to 1,000, 2,700, and 10,000 s light pulses, phase delays of 84.3 ± 8.2 min (p < 0.05), 134.0 ± 14.1 min (p < 0.01), and 168.5 ± 8.0 min (p < 0.01) were found, respectively. The duration response curve was fitted with a sigmoid function using the method of least squares (R^2^ = 0.998). These results show that the circadian system is capable of integrating UV light in a manner comparable to light in the visible spectrum [6, 9]. Collectively, the data are consistent with earlier reports that UV light can act as an important nonvisual stimulus in rodents [8, 10–18].

### UV Light Induces Sleep in *Opn4*^*+/+*^ and *Opn4*^*−/−*^ Mice

The contribution of UV light to the acute regulation of sleep was then examined. In *Opn4*^*+/+*^ mice (n = 5), a UV light pulse increased the amount of nonrapid eye movement (NREM) sleep by 93% (Figures 1D and 1E). The same UV light pulse administered to *Opn4*^*−/−*^ mice (n = 5) also significantly increased NREM sleep by 79%. REM sleep was increased by 166% in *Opn4*^*+/+*^ mice and by 98% in *Opn4*^*−/−*^ mice (see Figures S1A and S1B available online). The results show that UV is as effective as white light at inducing sleep [19] and that this response is independent of melanopsin.

Recent studies have demonstrated that melanopsin plays a dominant role in regulating the acute effects of white light on sleep induction [19–21]. The present study is the first to demonstrate that UV light exposure induces sleep in mammals and suggests that UV irradiance detection may be an important additional feature of non-image-forming responses to light in mice.

### SCN Electrical Activity

**Figure 2:**
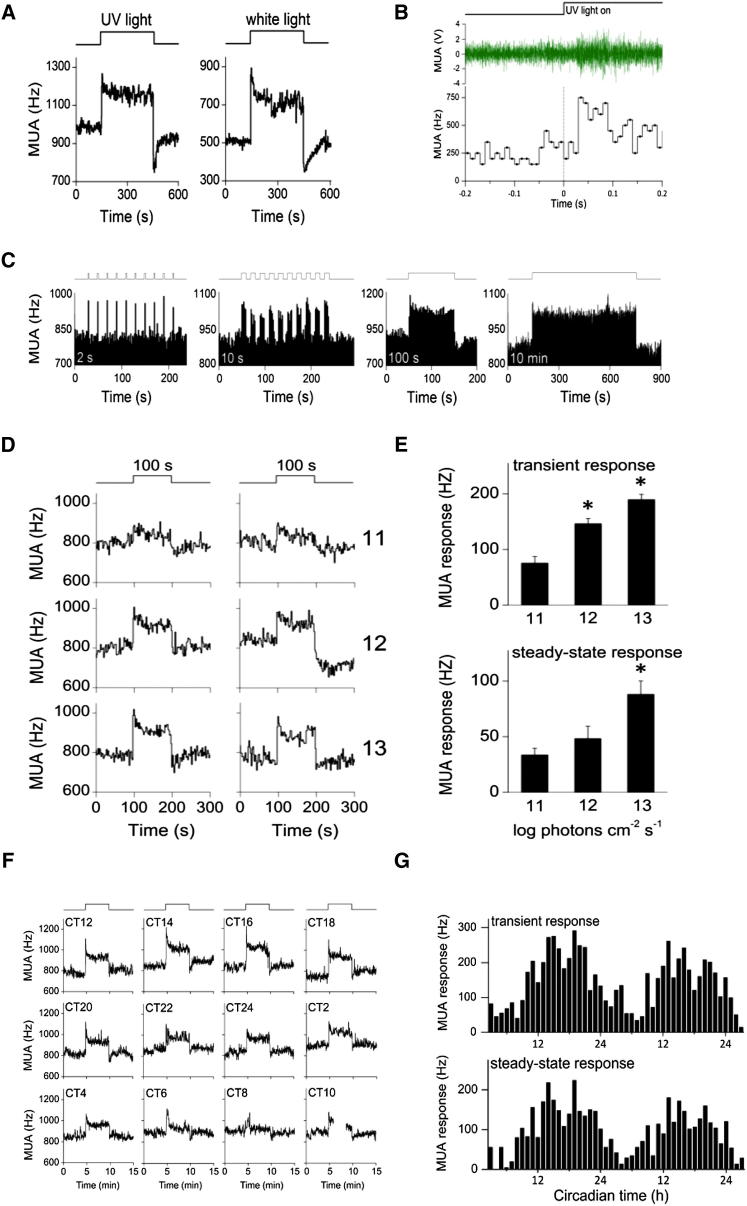
SCN Electrical Activity Responses to UV Light in Freely Moving Mice (A) Representative suprachiasmatic nucleus (SCN) multiunit activity (MUA) responses to a 5 min UV light or white light pulse. Bin size = 2 s. (B) Response latency to UV light. Time of lights-on is indicated by the step diagram. Green line shows a representative trace of multiunit activity in the SCN, with spike frequency above threshold shown below (bin size = 0.01 s). Vertical line indicates the time of UV onset (t = 0). SCN firing rate is increased in response to UV light, with a latency of 0.04 s. (C) MUA responses to UV light pulses of different durations, applied between CT14 and CT16. From left to right: 2 s lights-on, 18 s lights-off (10×); 10 s lights-on, 10 s lights-off (10×); 100 s lights-on; 10 min lights-on. Stimulus presentation is indicated by the step diagram above each plot. (D) Representative traces of SCN electrical activity to 100 s UV light pulses of different irradiances. Two examples are shown for each irradiance level. Log quanta/cm^2^/s is indicated on the right. (E) Summary of mean MUA response magnitudes ± SEM as a function of UV irradiance (11, 12, and 13 log quanta/cm^2^/s; n = 8, n = 8, and n = 7, respectively). Upper graph shows the transient response and the lower graph shows the steady-state level for the three irradiance levels. (F) Examples of SCN responses to 5 min UV light pulses at different times of the circadian cycle. (G) Offline analysis shows a clear circadian rhythm in transient “on-response” magnitude (upper graph) and in steady-state response magnitude (lower graph). The transient and steady-state responses were plotted versus circadian time over 48 hr. ^∗^p < 0.05. See also Figure S2.

UV light (365 nm) induced an increase in the suprachiasmatic nuclei (SCN) electrical discharge (Figure 2A). The UV light-activated response started with a transient overshoot at lights-on (“on-excitation”). During light exposure, electrical activity was elevated compared to baseline and the increased firing was maintained during exposure. At lights-off, a fast transient drop (“off-inhibition”) in SCN activity was observed, which gradually returned to baseline. Following UV lights-on, the response kinetics show a short latency of 30–40 ms (n = 30) (Figure 2B).

Highly elevated SCN discharge rates occur in response to UV light pulses as brief as 2 s (Figure 2C), consistent with studies showing chromatic sensitivity to flashes of light (0.1–1 s) [22]. Interestingly, with increasing stimulus durations, i.e., 10 s, 100 s, and up to 10 min, activated SCN cell populations maintained increased firing frequencies for the full duration of light exposure. The influence of irradiance on SCN electrical activity levels was investigated by exposure to 100 s UV light pulses with different irradiance levels ranging over 3 log units (11–13 log quanta/cm^2^/s, n = 7–8; Figure 2D). Increased stimulus irradiance resulted in more than a 2-fold increase in SCN firing response. Both the magnitudes of the transient on-excitation and the steady-state response were shown to be irradiance-dependent (11 versus 13 log quanta/cm^2^/s; p < 0.05; Figure 2E).

To determine the effect of circadian phase on UV light responses, mice were exposed to hourly 5 min UV light pulses over 24 hr (n = 1) or 48 hr (n = 2) (Figure 2F). Analyses of the on-excitation, as well as the steady-state response magnitude, showed a clear phase-dependent sensitivity of SCN electrical activity, with small responses during the day and large responses at night (Figure 2G). Such time-of-day effects have also been reported for white light [23]. Despite the responsiveness of the SCN to UV light during the subjective day (Figures 2F and 2G), no behavioral phase shifts are induced at this phase of the cycle (see phase response curve [PRC] in Figure 1B). This is consistent with the view that the signaling pathway mediating behavioral phase shifts is a postsynaptic event, downstream from the recorded membrane event [23].

### UV Light Responses in *Opn4*^*−/−*^ Mice

**Figure 3:**
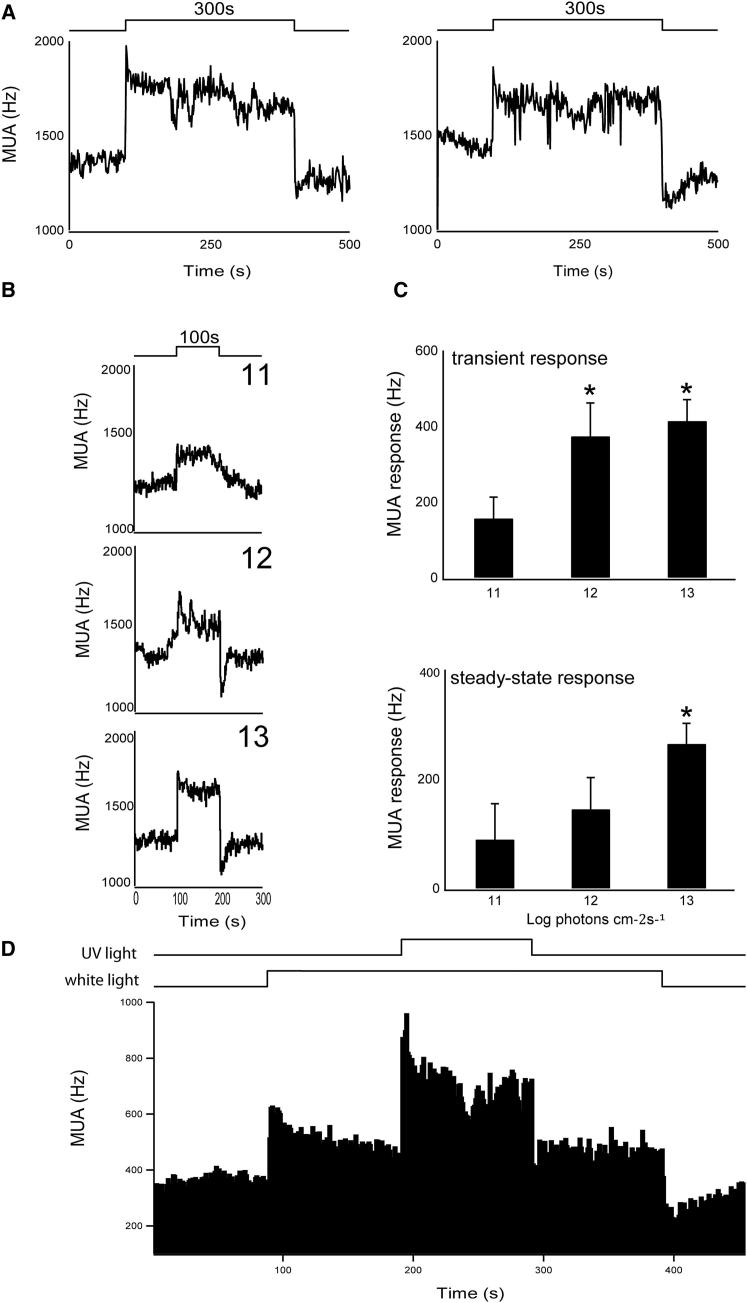
Responses to UV Light in the Absence of Melanopsin and under Photopic Light Conditions (A) Two representative traces of SCN MUA in response to 5 min UV light exposure in *Opn4*^*−/−*^ mice. MUA responses to UV light typically show a fast transient increase in spike frequency to lights-on, a sustained response during light exposure, and a fast transient decrease at lights-off. (B) Representative electrical responses to 100 s UV light exposure at three different irradiances. (C) Histograms showing mean MUA responses ± SEM as a function of UV irradiance (11, 12, and 13 log quanta/cm^2^/s; n = 4–7 per irradiance). Upper graph shows changes in the transient response and the lower graph shows changes in the steady-state response as a function of irradiance. (D) Representative SCN neuronal response to UV light under saturating white light in a wild-type mouse. An excitatory response of the SCN electrical discharge rate was induced by exposure to saturating white light. After 100 s, a 100 s UV light pulse was applied, which evoked a significant increment of 146% in the sustained SCN firing rate compared to saturating white light (n = 3). The light protocol is indicated by the bars above the graph. ^∗^p < 0.05. See also Figure S3.

We investigated whether the sustained UV light induced firing of SCN neurons is retained in melanopsin-deficient mice (*Opn4*^*−/−*^). SCN firing frequencies increased in response to UV light pulses (365 nm) of all durations tested (2 s, 10 s, 100 s, 10 min) (Figure S2). The firing pattern showed similar kinetics as described for C57BL/6 mice: fast transient “on-excitation” and “off-inhibition” responses, with an onset latency of 30–40 ms (n = 27); and sustained electrical discharge throughout light exposure (Figures 3A and 3B). The magnitude of the transient on-excitation as well as the steady-state response was irradiance dependent (11–13 log quanta/cm^2^/s, n = 4–7; Figure 3C), showing increased magnitude with increased irradiance (11 versus 13 log quanta/cm^2^/s; p < 0.05). These results are consistent with our observation that UV light is capable of evoking phase shifts in *Opn4*^*−/−*^ mice that are indistinguishable from those seen in wild-type littermate controls (Figure S1C).

### SCN Responses to UV Light Suggest a Role for UVS Cones

The results show that the sustained UV-driven SCN response occurs independently of melanopsin. The question is, how? The SCN receives its photic input via a small population of melanopsin-expressing retinal ganglion cells (pRGCs) [1, 24–27]. The pRGCs are in turn innervated indirectly by the rods and cones and via this route contribute to SCN light activation [28–31]. The very short latencies we observe in response to UV light are in close agreement with those previously shown for cone-mediated fast reaction times measured from pRGCs (30–40 ms [29]; 50–60 ms [31]) and differ from both rod-mediated response latencies (150 ms [29]) and melanopsin-mediated response latencies (>300 ms to minutes [1, 27, 29]). Thus, UV responses to light appear to be mediated by ultraviolet-sensitive (UVS) cones. However, we cannot exclude the possibility that the sustained component of the response might depend upon another class of photoreceptor, such as rods [32, 33]. To address whether the UV photosensitivity is mediated by UVS cones, we measured SCN responses to UV light superimposed upon a broad spectrum white light background. The white light, with no UV component, was used to saturate all photoreceptor classes, except the UVS cones (Figure S3A). Under these conditions, additional blue light failed to induce further increments of the electrical discharge rates (Figure S3B). Strikingly, however, when UV light was applied, significant increments in response magnitude were observed (p < 0.05) (Figure 3D). These responses showed both transients and sustained components. Our data are consistent with the view that UV light excites the UVS cones, which in turn are capable of adding to the saturated responses from other photoreceptors.

Our results show that UV light can elicit transient and sustained responses to light within the SCN. The high sensitivity to UV light both under scotopic and photopic conditions, the short response latency to UV light, the behavioral UV light responses in animals deficient of rods ([12]; this study) or melanopsin (this study), and other recent studies [30] all support the view that the UVS cones participate in pRGC signaling. Whether the capacity to encode steady-state irradiance is a special property of murine UVS cones, the response of pRGCs to UVS cone input, or even a dedicated input by UVS cones to a specialized subset of pRGCs remains to be determined.

### Phase-Shifting Responses to UV Light Are Cone Dependent

**Figure 4:**
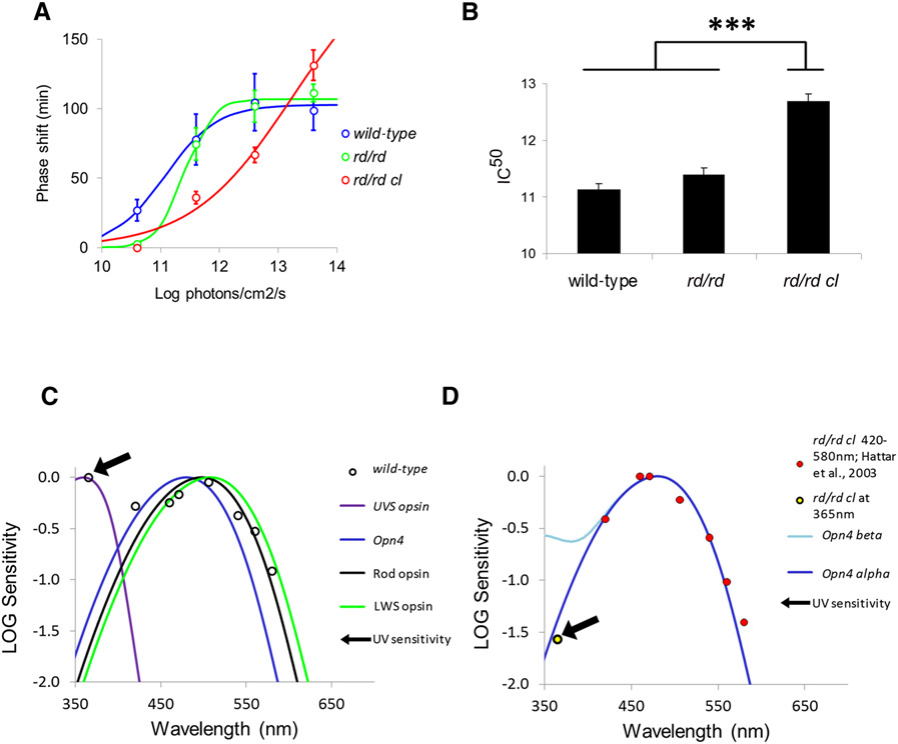
Phase-Shifting Responses to UV Light Are Cone Dependent (A) Irradiance response curves (IRCs) for phase-shifting responses ± SEM of wild-type C3H, *rd/rd*, and *rd/rd cl* mice to UV light (15 min pulse at CT16, 365 nm UV LEDs). (B) Sensitivity to UV light was assessed by IRC IC_50_. Mice lacking rods and retaining a reduced population of cones (*rd/rd*) show no reduction in UV sensitivity compared to wild-type controls. By contrast, mice lacking all rods and all cones (*rd/rd cl*) show a significant attenuation of UV sensitivity, with an IC_50_ 1.57 log units higher than controls (p = 1.80 × 10^−5^). Data points indicate mean ± SEM. (C) Action spectrum for circadian phase shifting in wild-type mice. Full irradiance response curves were constructed for eight monochromatic wavelengths (365, 420, 460, 471, 506, 540, 560, and 580 nm; see Figure S4A). Action spectrum data are plotted against the known photopigments of the mouse retina (UVS cone λ_max_ = 360 nm, Opn4 λ_max_ = 480, rod λ_max_ = 498 nm, LWS cone λ_max_ = 508 nm). (D) Action spectrum for circadian phase shifting in *rd/rd cl* mice. The irradiance response curve from Figure 4A was used to determine the sensitivity at 365 nm. For comparison, we have used our previously published data for 420, 460, 471, 506, 540, 560, and 580 nm [35]. Action spectrum data are plotted against the known absorption spectrum for the Opn4 photopigment (λ_max_ = 480), the only known photopigment remaining in the *rd/rd cl* retina. The full absorbance spectrum of any opsin/vitamin A visual pigment consists of an alpha band in the visual range (e.g., the alpha band λ_max_ for melanopsin is at 480 nm) and a smaller-amplitude and significantly shorter wavelength absorbance beta band (e.g., the beta band λ_max_ for melanopsin is at 345 nm). Normally only the alpha band is shown. Both the alpha and beta bands are shown in (D). Absorption by the beta band has been proposed as one mechanism whereby a photopigment with an alpha band in the visual range might still show relatively high sensitivity to UV light [36]. However, the strong match between the alpha band absorbance for melanopsin and UV sensitivity shown in (D) provides no evidence for beta band involvement.

To confirm the role of UVS cones, we assessed phase-shifting responses to light in retinal mutants/transgenics. UV responses are broadly similar between *rd/rd* mice (lacking rods but retaining a reduced population of cones [34]) and wild-type mice; however, at the lowest irradiance examined (10.6 log quanta/cm^2^/s), there is a significant attenuation in sensitivity (p = 0.03). These data suggest that normally, at low irradiances, rods contribute to the overall sensitivity to UV light. In contrast to *rd/rd* mice, the loss of all rods and cones in the *rd/rd cl* genotype results in a marked attenuation in UV sensitivity (Figure 4A). When plotted as IC_50_ (half maximal effective irradiance, based upon irradiance response curve [IRC] fitting to individual animals, in all cases R^2^ > 0.89), there is a significant difference in the IC_50_ between wild-type and *rd/rd cl* mice (∼1.5 log units, p = 1.8 × 10^−5^). These data demonstrate that the marked attenuation in UV sensitivity in *rd/rd cl* mice is concomitant with complete cone photoreceptor loss (Figure 4B). When the sensitivity of wild-type (Figure 4C; IRCs from Figure S4A) and *rd/rd cl* mice (Figure 4D; IRCs from Figure 4A and [35]) is compared across eight wavelengths, there are also notable differences. In wild-type mice possessing cones, rods, and melanopsin pRGCs, the sensitivity to UV light at 365 nm was equivalent to the sensitivity at 506 nm (Figure S4B). In contrast to wild-type mice, UV responses to light are markedly attenuated in the *rd/rd cl* genotype. The black arrows in Figures 4C and 4D indicate UV sensitivity in wild-type and *rd/rd cl*, respectively. This finding, loss of UV sensitivity in the absence of UVS cones, supports the hypothesis that UV responses to light in mice are mediated by UVS cones. Furthermore, the residual sensitivity observed in *rd/rd cl* mice matches precisely the short-wavelength limb of the absorption spectrum of melanopsin (Figure 4D).

### Conclusions

UV light exposure affects behavioral activity rhythms in an irradiance-, duration-, and time-dependent manner. UV light is also as effective as broad-spectrum white light in inducing sleep in mice [19]. Electrophysiological recordings from the SCN show acute responsiveness to UV light exposure. The response was characterized by fast transient components occurring at the light transitions and a sustained discharge that depends upon the level of illumination. Behavioral phase-shifting responses, sleep induction, and electrical responses to UV light were all preserved in the absence of melanopsin. Critically, mice lacking rods (*rd/rd*) show no attenuation in UV phase shifting at medium to high light intensities, whereas mice lacking rods and cones (*rd/rd cl*) show a significant attenuation of UV responses. Furthermore, the residual UV sensitivity in *rd/rd cl* mice is fully accounted for by the alpha absorption spectrum of melanopsin in the UV part of the spectrum. Collectively, our findings are consistent with the view that UV responses to light in mice are mediated by UVS cones. We also show that steady irradiance signaling can occur independently of melanopsin and that that UVS cones appear to play an important general role in detecting sustained ambient levels of light. This conclusion is supported by a recent study demonstrating a role for UVS cones in pupillary light constriction in mice [15].

## Supplemental Information

Document S1. Figures S1–S4 and Supplemental Experimental Procedures
