# Supplementary material for: Ultraviolet Light Provides a Major Input to Non-Image-Forming Light Detection in Mice
Source: Curr Biol. 2012 Aug 7;22(15):1397–402. doi: 10.1016/j.cub.2012.05.032 (PMC3414846; doi:10.1016/j.cub.2012.05.032)
Supplement: Document S1. Figures S1–S4 and Supplemental Experimental Procedures [file mmc1.pdf]

Current Biology, Volume *22*

Supplemental Information

Ultraviolet Light Provides a Major Input to Non-Image-Forming Light Detection in Mice

Floor van Oosterhout, Simon P. Fisher, Hester C. van Diepen, Thomas S. Watson, Thijs Houben, Henk Tjebbe VanderLeest, Stewart Thompson, Stuart N. Peirson, Russell G. Foster, and Johanna H. Meijer

Author Contributions

FvO and SPF contributed equally as first authors. RGF and JHM share senior authorship. TSW conducted UV IRCs. HvD, TH, HTV provided additional data for in vivo SCN recordings and ST collected phase-shifting behavioral data for the action spectra. FvO, SPF, HvD, TSW, SNP, RGF, JHM discussed and designed the experiments and wrote the paper.

Supplemental Inventory

Figure S1, related to Figure 1. REM sleep and phase shifting responses to UV light in *Opn4*+/+ and *Opn4*-/- mice are in correspondence with NREM sleep and phase shifting responses shown in Figure 1.

Figure S2, related to Figure 2. MUA responses to UV light pulses of different durations in *Opn4*-/- mice, these responses are similar to responses to UV light in wild-type mice presented in Figure 2C.

Figure S3, related to Figure 3. Control data of Figure 3D to show the effect of blue and UV light in a saturating white light background.

Figure S4, related to Figure 4. Phase-shifting responses over a range of wavelengths of light. These IRCs were used to generate the wild-type action spectrum in Figure 4A.

Supplemental Experimental Procedures. Full experimental procedures.

Supplemental References. References of Supplemental Experimental Procedures.

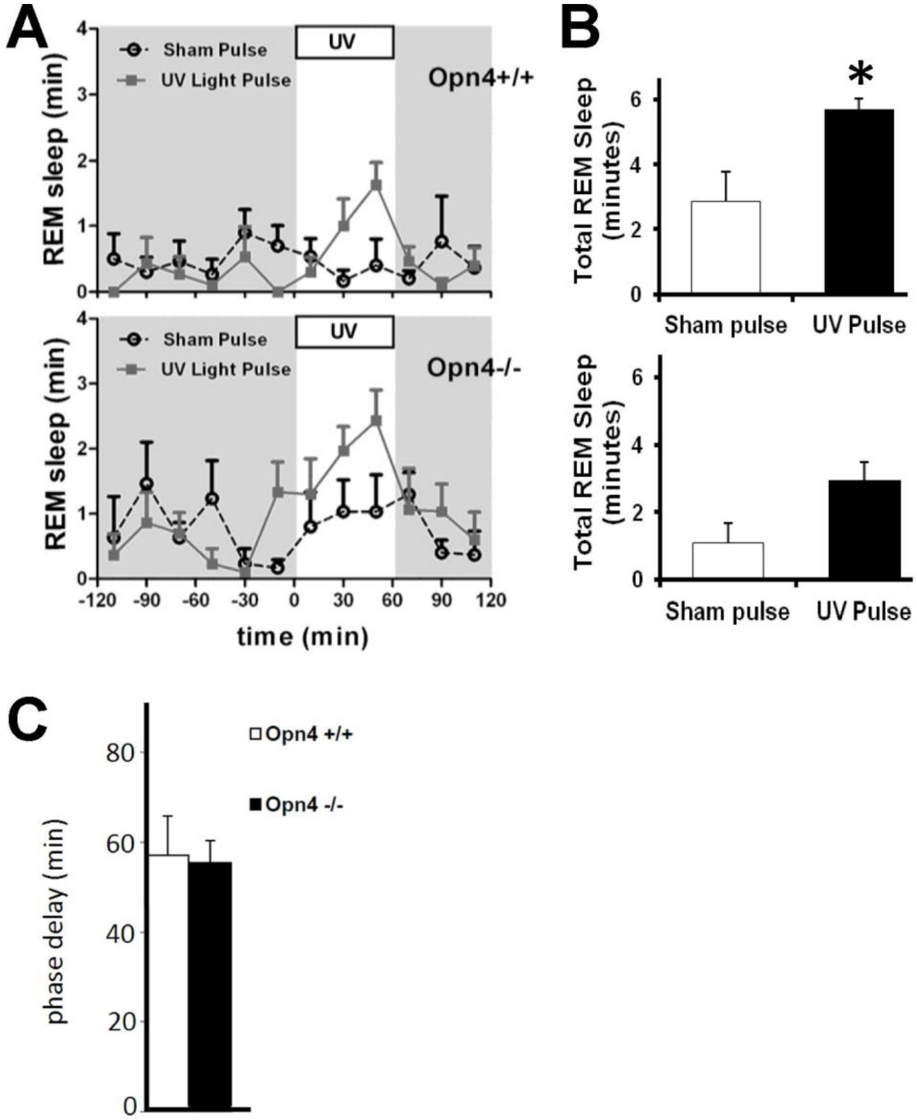

Figure S1, Related to Figure 1. Effect of UV Light on REM Sleep and Phase Shifting in *Opn4*$^{+/+}$ *and Opn4*$^{-/-}$ Mice

(A) Time course of REM sleep following UV light exposure, showing responses in *Opn4*$^{+/+}$ and *Opn4*$^{-/-}$ mice (n=5).

(B) Histograms summarizing changes in REM sleep in response to UV light exposure. UV light administered at ZT 16-17 results in a significant increase of REM sleep in *Opn4*$^{+/+}$ mice. Whilst REM sleep also appeared to increase in *Opn4*$^{-/-}$ animals, this was not statistically significant. * indicates P<0.05.

(C) *Opn4*$^{+/+}$ and *Opn4*$^{-/-}$ mice were maintained under a light/dark cycle of 12h/12h (L:D 12:12) with free access to running wheels. Once fully entrained, mice were exposed to a 15-min light pulse of UV light (11.8 log quanta/cm2/s) at Zeitgeber Time 14 (n =9 per genotype). After the pulse, mice were placed in constant darkness for an additional 10 days to determine the phase shift in behavioral activity. Exposure to UV light produced an equivalent phase delay in *Opn4*$^{+/+}$ and *Opn4*$^{-/-}$ mice. Data are presented as mean values (± SEM).

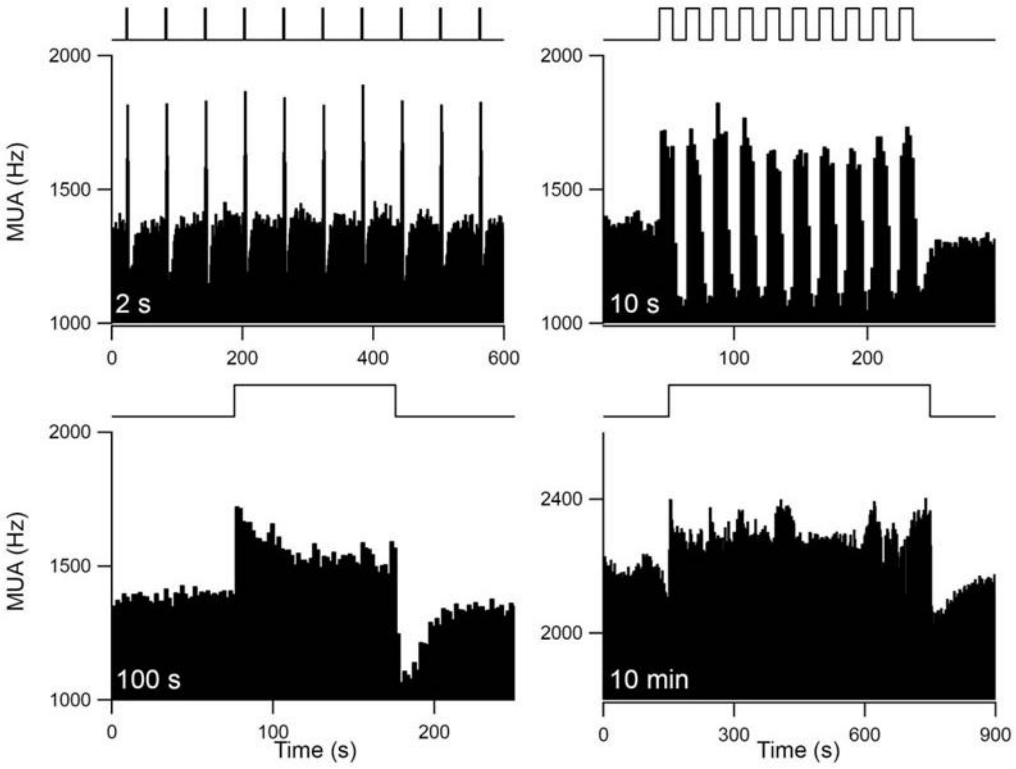

Figure S2, Related to Figure 2. SCN Electrical Responses to UV Light Pulses of Different Durations in Melanopsin-Deficient (*Opn4*$^{-/-}$) Mice

Upper left: 2s lights on, 58s lights off (10x); Upper right: 10s lights on, 10s lights off (10x); Lower left: 100s lights on; Lower right: 10 min lights on. Bin size is 1s. MUA = multi-unit activity. Stimulus presentation is indicated by the step diagram above each plot.

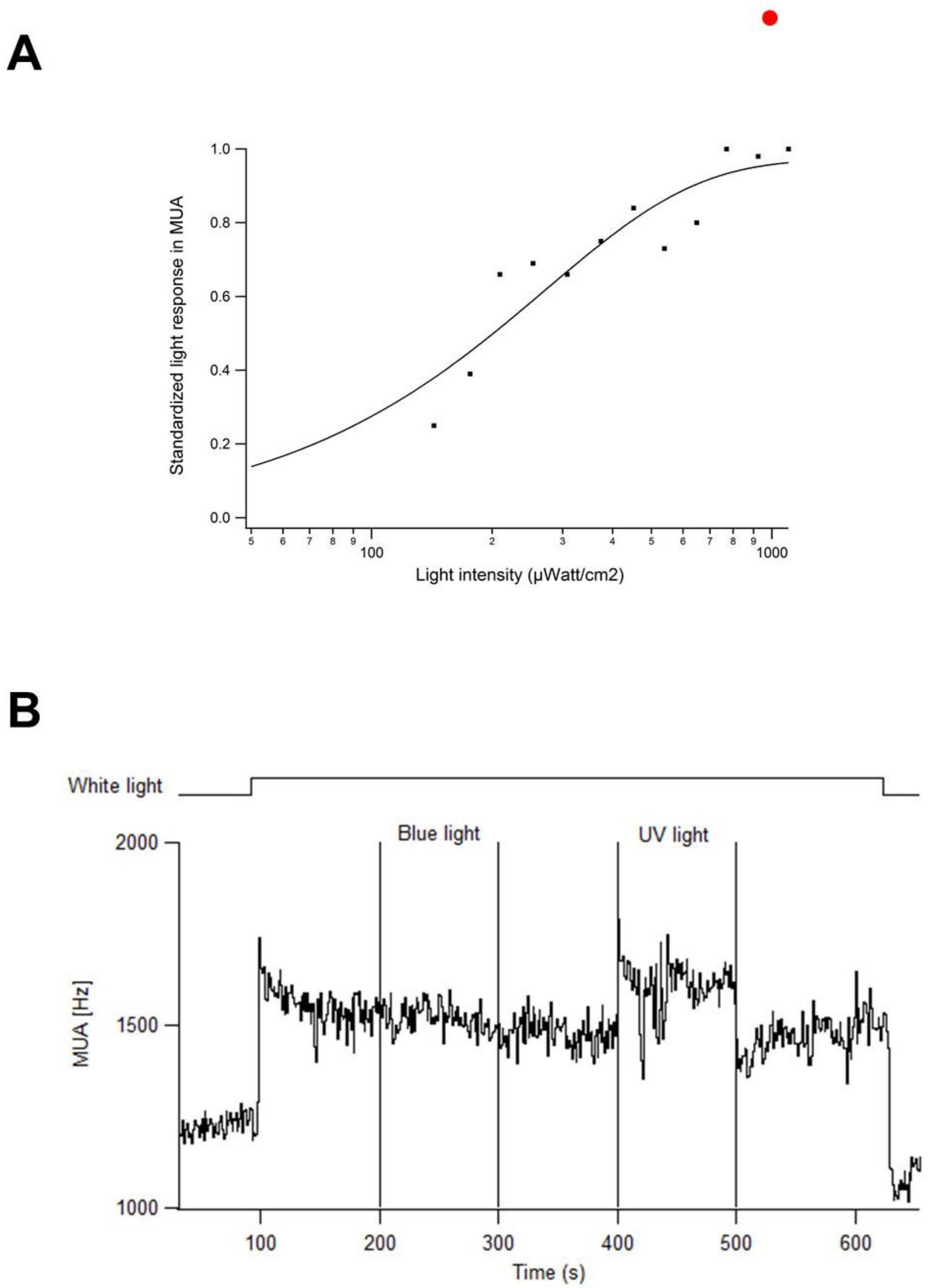
A
1.0
0.8
0.6
0.4
0.2
0.0
Standardized light response in MUA
5
6
7
8
9
100
2
3
4
5
6
7
8
9
1000
Light intensity (µWatt/cm2)
B
White light
Blue light
UV light
2000
1500
1000
MUA [Hz]
100
200
300
400
500
600
Time (s)

Figure S3.

Figure S3, Related to Figure 3. Responsiveness to UV Light in a Saturating White Light Background

(A) Irradiance response curve in SCN electrical activity for white light. The sustained light response in SCN electrical activity is plotted against the light intensity. The maximal light response was normalized to 1. The relationship between irradiance and response magnitude in SCN electrical activity is fitted with a slope sigmoid dose response function. The response to the three highest light intensities did not show significant differences ($p > 0.05$). The red dot indicates the significant mean sustained increment in SCN electrical activity in response (146 %) to UV light on top of the sustained white light-induced response.

(B) SCN electrical activity in response to saturating white light (light intensity; 1100 µWatt/cm^2). An additional blue light pulse (13 log quanta/cm^2/s; λ=465 nm) did not lead to significant increment in discharge rate (n=4, in two wild-type mice) ($p > 0.05$). In contrast, an additional UV light pulse (13 log quanta/cm^2/s; λ=367 nm) caused a significant increment in response ($p < 0.05$). Data were analyzed by comparing baseline discharge levels (last 25 sec before blue and UV light respectively), with the first 25 sec during the pulse using a two-tailed unpaired Student's t-test.

A

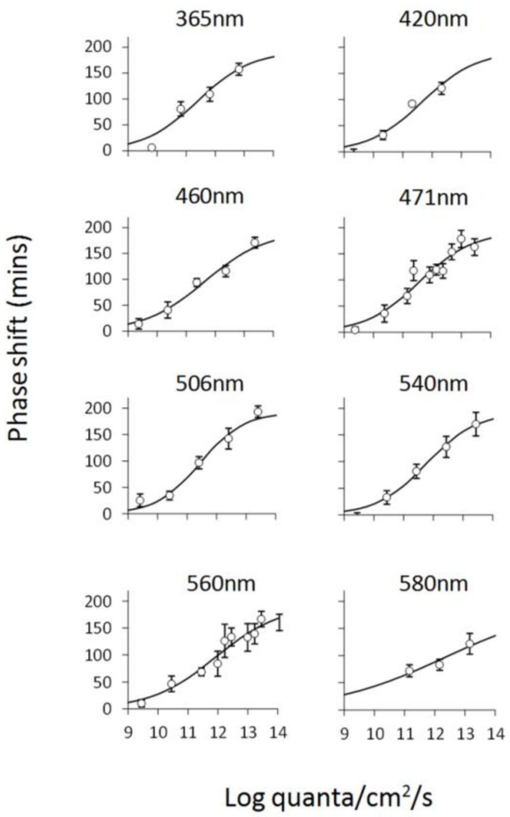

B

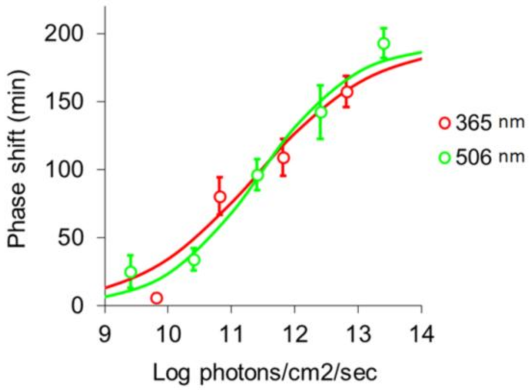

Figure S4, Related to figure 4. Irradiance Response Curves (IRCs) for Phase-Shifting Responses in Wild-Type Mice over a Range of Wavelengths

(A) The relationship between irradiance and phase shift magnitude (mean and standard error) is fitted with a variable slope sigmoid dose response function. Maximum phase shift obtained was 192.1mins. These IRCs were used to generate a wild-type action spectrum (Figure 4A) at eight monochromatic wavelengths: 365, 420, 460, 471, 506, 540, 560 and 580nm.

(B) Statistical analysis indicates that the IRC for wild type phase-shifting responses (Figure S4A) to 365nm light was not significantly different from responses at maximal sensitivity (506nm IRC) ($F_{2,48}$=0.41, P=0.668). The IRCs were tested using a modified F-test as described previously [5].

Supplemental Experimental Procedures

Animals

Adult male C57BL/6 mice (Harlan, Horst, The Netherlands) (aged 4-12 months) were used to construct behavioral phase and duration response curves for UV-light. Phase shifting studies were performed in male *Opn4*$^{+/+}$ and *Opn4*$^{-/-}$ mice on a C57BL/6 x S129 background (aged 4-8 months). In vivo recordings of multiunit activity from the SCN were performed in male C57BL/6 mice (aged 3-12 months) as well as in male *Opn4*$^{+/+}$ and *Opn4*$^{-/-}$ mice (aged 3-6 months). Telemetry studies were performed in male *Opn4*$^{+/+}$ and *Opn4*$^{-/-}$ mice (aged 4-8 months). Adult male *rd/rd* and *rd/rd cl* mice were used to construct the irradiance response curves. All animals were on a C3H background and were 80-100 days old at the beginning of the phase shift experiment. Male C3H wild-type (not carrying the *rd* mutation) controls were age-matched.

Mice were individually housed in cages and isolated from possible confounding external stimuli in light-tight ventilated chambers. Prior to the start of all experiments, mice were entrained to a 12h:12h L:D cycle for a minimum of 14 days. Light onset was designated as Zeitgeber time (ZT) 0 and dark onset as ZT12. Light-dependent resistors connected to a ClockLab interface (Actimetrics, Wilmette, IL, USA) were used to record the light/dark conditions at all times. Ambient temperature was maintained at 20 ± 2 °C and food and water were available *ad libitum*. All aspects of animal work were carried out under Home Office license in accordance with the Animal (Scientific Procedures) Act 1986 (UK), and under the approval of the Animal Experiments Ethical Committee of the Leiden University Medical Centre (The Netherlands).

Circadian Activity Monitoring

Techniques and protocols for measuring circadian phase shifts to discrete light pulses using the phase of onset of circadian wheel running are well established [1]. Mice were individually housed in cages fitted with running wheels and the presence of wheel-running activity was automatically recorded in 1-min bins by the ClockLab data acquisition system (Actimetrics, Wilmette, IL, USA). Animals were placed in continuous darkness prior to exposure to a phase delaying light pulse. The magnitude of the steady-state behavioral phase shift was determined by fitting straight lines through the activity onsets before and after the light pulse. The difference between the two lines on the day of the light pulse determined the magnitude of the phase shift. Because of transients, the activity onsets on the first three days after the light pulse were excluded from analysis.

Phase Response Curve and Duration Response Curve to UV Light

C57BL/6 mice were entrained to 12:12 LD cycles and released into constant darkness. UV light pulses (365nm; $\lambda_{1/2max}$ 9 nm; 12.9 log quanta/cm^2/s) were applied on the 7th day in DD by transferring the animals to the UV-light set-up within the same room. The phase response curve was constructed from 45 min light pulses applied at different times of the day. The timing of light exposure was calculated based on an animal's individual activity pattern in DD. Following each light pulse, animals were re-entrained to a 12:12 LD cycle before this process was repeated, with a maximum of two light pulses per animal. The duration response curve was constructed from light pulses of different durations (2s, n=4; 10 s, n=6; 100s, n=7; 1000s, n=10;

2700s, n=5; 4000s, n=2; 10000s, n=4) applied at CT14-16 (the time of maximal delays, see Figure 2B).

EEG/EMG Sleep Recordings in *Opn4*$^{+/+}$ and *Opn4*$^{-/-}$ Mice in Response to UV Light

To determine if UV light is capable of modulating sleep in the same manner as white light, we also evaluated sleep induction in response to nocturnal UV light exposure. Opn4$^{+/+}$ and Opn4$^{-/-}$ mice anaesthetized under isoflurane (1-3%) were implanted with EEG/EMG telemetry transmitters (TL11M2-F-20, EET, Data Sciences International, St. Paul, MN, USA). Buprenorphrine (Vetergesic 0.015 mg/kg, Alstoe Animal Health, UK) was administered prior to surgery to minimize post-operative pain and temperature was maintained throughout using a heated mat (Habistat, UK). The body of the implant was positioned subcutaneously on the dorsum with EEG electrodes positioned on the cortical surface at following coordinates (1 mm anterior to bregma and 1 mm lateral to the central suture; 3 mm posterior to bregma and 3 mm lateral to the central suture). Electrodes and leads were secured in place using dental adhesive (Reliance, IL, US). EMG electrodes were inserted bilaterally into either side of the musculus cervicoauricularis. Mice were allowed at least 3 weeks to recover before studies were undertaken. All implanted mice remained healthy and gave clear EEG/EMG recordings allowing the discrimination of sleep/wake stages for >4 months. EEG and EMG data were transmitted to a radio receiver (RPC-1, Data Sciences International, St. Paul, MN, USA) placed underneath each cage. Signals were then routed via a data exchange matrix to a PC running Dataquest A.R.T. software (version 3.01). The EEG and EMG data were continuously sampled using DSI Dataquest Gold acquisition software (DSI) at 500 Hz, with a 100 Hz filter cut-off. EEG and EMG signals were band-pass filtered (0.5-35 Hz for EEG and 80-100 Hz for EMG) and sleep/wake stages were scored offline as wakefulness, non-rapid eye movement (NREM) sleep and rapid eye movement (REM) sleep in 10 sec epochs using a semi-automated approach. The sleep scoring procedure consisted an initial automated step using SleepSign® software (Kissei Comtec, Nagano, Japan), followed by a review of all epochs by an experienced sleep scorer. During a baseline period (day 1) *Opn4*$^{+/+}$ and *Opn4*$^{-/-}$ mice implanted with EEG/EMG telemetry transmitters housed in a light tight chamber were left undisturbed in the usual light/dark conditions. On the following day (day 2), a 1-hour 363nm light pulse was administered at ZT16. EEG and EMG were recorded continuously for a period of four hours from ZT14-ZT18 on both experimental days. Time course analysis and the total NREM and REM sleep were calculated for the 1 hour light pulse vs. the 1-hour sham period for each genotype.

Statistical Analysis

Statistical analysis was performed using GraphPad Prism software (GraphPad) or OriginV7 software (OriginLab, Northampton, MA, USA). Significant differences between groups were determined using either two-tailed unpaired Student's t-test or one-way ANOVA, followed by Bonferroni's *post-hoc* test. Values were considered significantly different where $p < 0.05$.

Electrophysiology Recordings

Procedures for in vivo recordings of multiunit activity (MUA) from SCN neurons have been described previously [2]. Using a stereotactic instrument, tripolar stainless steel micro-electrodes (Plastics One, USA) were implanted in mice (minimum age 12 weeks, 20-30 gram)

under Hypnorm/Dormicum anesthesia. Two twisted electrodes (Polyimide-insulated; bare electrode diameter 0.125 mm) for differential recording were aimed at the SCN (coordinates: 0.46 mm posterior to bregma, 0.14 mm lateral to the midline, 5.2 mm ventral to the surface of the cortex, under a 5-degree angle in the coronal plane [3], and a third uncoated reference electrode was placed in the cortex. After recovery, the animals were connected to the recording system with a counterbalanced swivel system enabling the animals to move freely during the measurements. The electrical signal was amplified and bandwidth filtered (0.5 - 5kHz). Window discriminators were used to convert action potentials into digital pulses that were counted in 2 sec epochs (CircaV1.9 custom made software) and stored for offline analysis.

To obtain uniform illumination levels, recordings were performed in a half-sphere (diameter 30 cm) coated with high reflectance paint (Barium Sulphate; Labsphere WRC-680). At the top of the sphere an opening (diameter 5 cm) was created for the connecting swivel system. The sphere was illuminated by monochromatic UV light using high power LEDs (NCCU033, Nichia, Japan) positioned in a circle at the upper part of the sphere, and a baffle prevented the animal from looking directly into the light. Within the sphere a UV-transparent perspex cylinder was used to house the animal. The actual wavelength and light irradiances were measured at a fixed position on the floor of the cage using a calibrated spectrometer (AvaSpec2048, Avantes, The Netherlands). Irradiance levels were manually regulated using a current source and the timing of the applied light pulses was computer controlled. All light pulses were applied against a dark background. The actual wavelength was 365 nm and half-maximal bandwidth ($\lambda_{1/2}$) was 9 nm. To characterize the electrical response pattern of the SCN, UV light pulses of various durations (ranging from 2 sec to 10 min at constant irradiance of 12.9 log quanta/cm^2/s) and intensities (ranging from 11 to 13 log quanta/cm^2/s; 100 sec pulses) were applied between CT14-16. The absolute magnitude of light responses between animals was different, due to differences in spike recording settings and/or differences in recording location within the SCN (i.e. highly innervated versus less innervated area). For the quantification of irradiance responses, mean changes in discharge rate as compared to baseline levels were calculated. The baseline level was defined as the average firing rate of the last 100 sec before lights on, the transient on-excitation discharge level was determined by the firing rate of the first 2 sec epoch after lights on, and the steady state discharge level was quantified as the average firing rate during the entire period of light exposure (excluding the first 50 sec due to the transient response). To investigate responsiveness of the SCN to UV light as a function of circadian time, 5-min light pulses of constant irradiance (12.9 log quanta/cm^2/s) were applied every hour. The circadian time of the light responses was calculated per day on the basis of the onset of behavioral activity as recorded by a passive infrared sensor.

To investigate the responses to the light pulses in more detail, the electrophysiological signals of SCN activity were digitized at 25 kHz using Spike2 hardware and software (Cambridge Electronic Design) and stored for off-line analysis. The digitized recordings were imported into MATLAB as 'waveform data', including data from light and movement sensors, using parts of the sigTOOL SON Library (http://sigtool.sourceforge.net). Imported waveform data were triggered at fixed voltage amplitude settings, and time and amplitude of these action potentials were used for the analysis. To investigate the latency of the neuronal response to light onset, digitized action potentials were counted in 0.01s bins. For a detailed analysis of population activity, a baseline recording (100s before light pulse) was used to create a spike amplitude

histogram. On the basis of this amplitude histogram, thresholds were set in such a way that the average number of counts within each threshold window was equal. Threshold windows were non-overlapping and started above noise level. Action potentials were counted within each step of set thresholds for the remainder of the recording. The measurement of SCN sub-populations on the basis of baseline electrical activity levels leaves the neuronal response after baseline as a free parameter (see [4]), and enables comparisons between different, approximately equal-sized, populations of neurons. The use of these datasets provides insight into firing responses of small populations of cells. Action potentials were counted in 1s bins.

Histology

At the end of each recording, tissue was collected for the purpose of histological verification of the electrode location. The recording site was marked by passing a small electrolytic current through the electrode to deposit iron at the electrode tip, which was stained blue by immersing the brain in a potassium ferrocyanide containing fixative solution. The brain was sectioned coronally and stained with neutral red for microscopic reconstruction of the recording site. Recordings from outside the SCN were excluded from the final analysis.

UV Irradiance Response Curves

All control C3H, *rd/rd* and *rd/rd cl* were entrained to 12/12 LD then placed in DD for 2 weeks prior to the initial UV light pulse. Light pulses at 365 nm were given weekly for 15 minutes in a separate pulsing chamber at CT16 using a custom-made light source consisting of 5 UV LEDs (NCCU033, Nichia, Japan). Excel (Microsoft) was used to fit a four parameter sigmoid curve based upon the method of least squares, as described previously [5].

Supplemental References

1. Albrecht, U., and Foster, R.G. (2002). Placing ocular mutants into a functional context: a chronobiological approach. Methods *28*, 465-477.
2. Meijer, J.H., Watanabe, K., Schaap, J., Albus, H., and Detari, L. (1998). Light responsiveness of the suprachiasmatic nucleus: long-term multiunit and single-unit recordings in freely moving rats. J Neurosci *18*, 9078-9087.
3. Paxinos, G., and Franklin, K.B.J. (2001). The mouse brain in stereotaxic coordinates 2nd edition Edition, (San Diego: Academic Press).
4. vanderLeest, H.T., Rohling, J.H., Michel, S., and Meijer, J.H. (2009). Phase shifting capacity of the circadian pacemaker determined by the SCN neuronal network organization. PLoS One *4*, e4976.
5. Peirson, S.N., Thompson, S., Hankins, M.W., and Foster, R.G. (2005). Mammalian photoentrainment: results, methods, and approaches. Methods Enzymol *393*, 697-726.
